# HIV disclosure in the social networks of adolescents with HIV in Cape Town, South Africa

**DOI:** 10.3389/frph.2025.1545039

**Published:** 2025-05-21

**Authors:** Marta I. Mulawa, Frances Hung, Nicole J. Phillips, Mark F. Austin, Richard Barfield, Hui-Jie Lee, Heather J. Zar, Dan J. Stein, Jacqueline Hoare

**Affiliations:** ^1^ School of Nursing, Duke University, Durham, NC, United States; ^2^ Duke Global Health Institute, Duke University, Durham, NC, United States; ^3^ Department of Biostatistics and Bioinformatics, School of Medicine, Duke University, Durham, NC, United States; ^4^ Department of Psychiatry and Mental Health, University of Cape Town, Cape Town, South Africa; ^5^ Neuroscience Institute, University of Cape Town, Cape Town, South Africa; ^6^ Department of Statistics, NC State University, Raleigh, NC, United States; ^7^ Department of Paediatrics and Child Health, University of Cape Town, Cape Town, South Africa; ^8^ Unit on Child & Adolescent Health, SA Medical Research Council, Cape Town, South Africa; ^9^ Unit on Risk & Resilience in Mental Disorders, SA Medical Research Council, Cape Town, South Africa

**Keywords:** HIV, disclosure, adolescents, youth, South Africa, network analysis

## Abstract

**Background:**

Little is known about HIV status disclosure within the social networks of adolescents with perinatally-acquired HIV (APHIV) in sub-Saharan Africa, where HIV prevalence is high and stigma surrounding HIV is pervasive. This study employed egocentric network analysis to characterize HIV disclosure in the social networks of APHIV in Cape Town South Africa. Associations between HIV disclosure and characteristics of APHIV (egos), their relationship to network members (alters), and features of the social network, including ego's network size and alter's centrality within the network, were also examined.

**Methods:**

Network data were collected from 58 APHIV (mean age 14.3 years; 52% female) using a customized touchscreen-based assessment, providing data on 349 ego-alter relationships. Associations with HIV disclosure within the ego-alter pairs were evaluated by adjusted odds ratios (AOR) from multivariable logistic regression with generalized estimating equations, clustering by ego.

**Results:**

Among all ego-alter pairs identified, 36.4% (127/349) were disclosed to by egos. Odds of disclosure did not differ based on the ego's gender or viral suppression. Odds of disclosure were significantly higher when alters were presumed by the ego to have HIV [AOR 6.26; 95% confidence interval (CI): 2.36–16.58, *p* < 0.001], were more trusted by the ego (AOR 2.84; 95% CI: 1.14–7.06, *p* = 0.03), or were older than the ego (1-year difference AOR 1.04; 95% CI: 1.02–1.07, *p* < 0.001). Increasing alter's centrality by 0.01 was associated with an increased odds of disclosure (AOR 1.02; 95% CI: 1.002–1.03, *p* = 0.03), though overall network size was not associated with increased odds of disclosure. The effect of the ego's emotional support network size on disclosure, however, depended on the type of ego-alter relationship.

**Conclusions:**

HIV status disclosure was low in the social networks of APHIV. Our study found that disclosure was not influenced by network size, gender, or viral suppression but rather by factors related to interpersonal trust and shared experience, such as perceived HIV status of alters, network-level centrality and emotional support network size. The study further highlights the benefits and future potential of using social network data collection and analysis methods to better understand HIV disclosure among adolescents.

## 1 Introduction

Adolescents with perinatally-acquired HIV (APHIV) face unique challenges in sub-Saharan Africa, where HIV prevalence is high, and stigma surrounding HIV remains pervasive (1, 2). In 2020, approximately 1.8 million adolescents were living with HIV, representing 5% of all people living with HIV (3). Importantly, adolescents living with HIV are the only age group that has not seen significant declines in AIDS-related deaths (4) and adolescents repeatedly demonstrate lower levels of retention in HIV care and adherence to ART compared to older adults (5). These outcomes are thought to be due to numerous challenges experienced by adolescents, including HIV-related stigma, limited youth-friendly healthcare services, and the complexities of navigating a lifelong, stigmatizing chronic illness during a period of significant developmental, psychological, and social changes (6–8).

Disclosure of one's HIV status may open avenues to receiving social support to cope with the challenges unique to living with HIV. Among adults living with HIV in sub-Saharan Africa, disclosure has been linked to improved adherence to antiretroviral therapy (ART) and greater levels of retention in HIV care (9). It has also been shown to be associated with use of maternal health services and services for prevention of mother-to-child transmission of HIV (PMTCT) (10). Disclosure can also play an important role in preventing the onward transmission of HIV by facilitating HIV testing of sexual partners and linking partners to pre-exposure prophylaxis (PrEP). Less is known about the advantages of disclosure for APHIV (11, 12).

Despite its benefits, disclosure comes with the potential risk of stigma, rejection, isolation, and mental distress. These negative consequences may arise from entrenched societal attitudes and misconceptions surrounding HIV, which perpetuate fear and stigma in sub-Saharan Africa (13). Fear and anticipation of these risks is known to limit disclosure (14), and individuals with HIV must balance these potential risks with potential benefits when deciding to disclose their status to somebody new. For adolescents, who may be newly navigating social relationships and seeking peer acceptance, making disclosure decisions may be particularly challenging. Indeed, disclosure-related anxiety was identified as a key theme in a systematic review focused on stigma-related challenges among children and adolescents with HIV, including multiple studies from sub-Saharan Africa (2).

Notably, APHIV have been shown to have lower rates of disclosure compared to adolescents who acquired HIV more recently (15). Disclosure research among APHIV in sub-Saharan Africa has largely focused on examining correlates of disclosure to the adolescents/children who acquired HIV perinatally (16, 17), rather than examining APHIV's disclosure of their status to others (18). In non-African settings, increased disclosure among APHIV has been associated with older age, increased disclosure intentions, and earlier age of learning one's serostatus (19). Qualitative research with APHIV in South Africa has found that APHIV rarely disclose their status due to lack of trust and their fears of further unwanted disclosure, leaving their relationships feeling disconnected and superficial (20). While such fear of HIV status disclosure and stigma have been generally identified in studies of APHIV (6), little remains known about how APHIV navigate the disclosure process within their social networks.

Social networks, which refer to the informal patterns of relationships between individuals, can offer valuable insights into social dynamics, including disclosure and social support. Research has shown that social networks play a critical role in shaping health behaviors among adolescents (21). Social network analysis, a set of methods that focuses on analyzing the patterns of linkages between individuals (22), has been used to deepen our understanding of disclosure of same-sex behaviors among MSM's social networks (23). Notably, this research could further our understanding of disclosure in among APHIV, since existing research on disclosure has yet to explore how the structural and functional aspects of social networks shape HIV disclosure in this population.

Methodological barriers, such as the time-intensive nature of traditional social network assessments, have hindered the collection of comprehensive network data. Advances in technology have addressed many of these challenges, making it feasible to collect detailed social network data in a less burdensome and more efficient and youth friendly manner. Touchscreen-based tools and graph databases, for example, allow study participants and researchers to map social networks with reduced participant fatigue (24). These innovations enable the exploration and analysis of social support, trust, and engagement within networks, which are particularly relevant for APHIV as they navigate disclosure decisions.

This study leverages such technological advancements to explore disclosure within egocentric networks [i.e., personal networks based on the sets of ties that surround each participant (25)] among APHIV in Cape Town, South Africa. The purpose of our study was to characterize HIV disclosure in the social networks of APHIV and to examine the associations between HIV disclosure and characteristics of APHIV (egos), their relationship to network members (alters), and features of the social network, including ego's network size and alter's centrality within the network.

## 2 Methods

### 2.1 Procedures

This study was nested within the neurological sub-study of the Cape Town Adolescent Antiretroviral Cohort (CTAAC), a prospective cohort study of APHIV established on ART and matched HIV negative controls in Cape Town, South Africa (26, 27). Adolescents aged between 9 and 14 years old living with perinatally-acquired HIV were recruited for the CTAAC cohort from public sector health care services from across Cape Town. Participants were recruited, between August 2013 through April 2015 from seven clinics providing routine ART services across Cape Town and followed at six-month intervals for study assessments. The inclusion criteria were having been on ART for at least 6 months, knowing their HIV status, and having the ability to follow-up, selected to recruit a generalizable sample of APHIV on ART in the study area. Exclusion criteria included having an uncontrolled medical condition, an identified central nervous system condition, a history of head injury, or a history of perinatal complications or neurodevelopment disorder not attributed to HIV. Adolescents provided assent to participate and their primary caregiver provided written informed consent. A total of 122 APHIV (aged 9–12 years old at baseline) were enrolled in the neurological HIV sub-study (28), and these participants underwent neuroimaging, neuropsychological and mental health assessment at baseline and 36 months. At the 36-month visits, network measures were assessed among 61 APHIV, aged 12–15 years old, who were sampled consequently once the network sub-study was approved. No participants declined to participate in the network assessment.

To collect egocentric network (i.e., personal network) data, we customized a network data capture instrument based on an existing tool, netCanvas (24). NetCanvas was designed to efficiently enumerate egocentric social networks with less burden placed on participants. The tool utilizes touchscreen technology and is designed to be user-centered, with participants completing the assessment themselves with the aid of a research assistant. Our team customized netCanvas for use with isiXhosa-speaking participants in South Africa. The original netCanvas software has continued development and has now become the open-source project Network Canvas (29).

Our customized tool collected egocentric social network data for each participant through the following phases: name generating, name interpreting, and edge generating. In the name generating phase, participants were asked to provide names or nicknames for members of their social networks. Specifically, participants were asked “Who are the people you are closest to? That is, people you see or talk to on a regular basis and share your personal thoughts and feelings with?” To add an individual, participants were instructed to enter the person's first name (or pseudonym), last name (or pseudonym), nickname (optional; if not entered, the app would display initials of first and last name), age, and role (check all that apply including friend, family/relative, romantic partner, acquaintance, and other). Once the participant was done adding people to their network, they were asked a series of additional name generating prompts, including: “If you wanted to talk to someone about things that are very personal and private, is there anybody else you could talk to?” Other name generating prompts included people not already mentioned that the participant could borrow money from, ask advice about health issues or advice about their treatment, friends met at their clinic, and individuals the participant has had sex with.

In the name interpreting phase, respondents provided additional information about these individuals (e.g., demographics, level of trust, HIV disclosure, social support received). Specific questions asked are descried in the Measures section on Alter-level Network Characteristics below. To provide data on social support, participants were instructed to tap on all the people in their network who had provided various types of instrumental social support, emotional social support, and medical-specific social support. Participants were also asked to identify the network members who, to their knowledge of the ego, were living with HIV. Participants were assisted by research assistants who were fluent in both English and isiXhosa.

**Figure 1:**
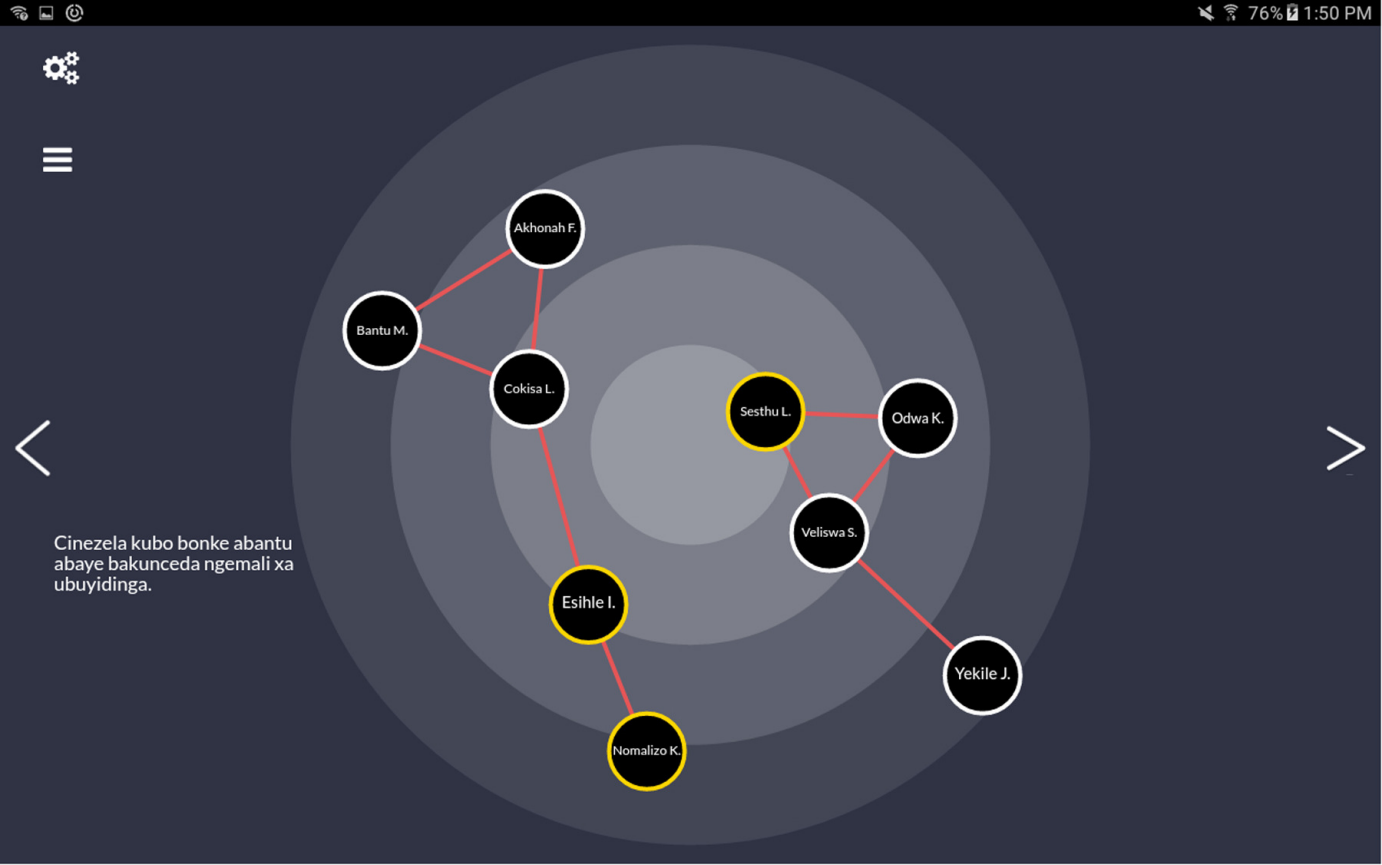
Screenshot of customized tablet-based social network data collection tool depicting a simulated network with 9 alters. HIV disclosure is indicated by yellow highlighting and edges (i.e., social ties between two network members) are denoted as red lines. The data shown are simulated and do not represent real individuals or relationships.

In the edge generating phase, participants were asked to connect any two network members who are close to each other or have spent time together in the past six months (Figure 1). Network and participant data were stored on encrypted tablets and uploaded to a secure server. All study procedures, for both the larger CTAAC trial as well as this Social Network study, were approved by the Institutional Review Board at Duke University and the Human Research Ethics Committee of the Faculty of Health Sciences at the University of Cape Town.

### 2.2 Measures

#### 2.2.1 Demographic characteristics and clinical measures

As part of the CTAAC study, all participants completed self-reported assessments and provided blood samples at all study visits to obtain clinical data. For this study, demographic measures of interest included participant gender and age at the 36-month assessment. Additionally, HIV-related clinical data collected at the 36-month CTAAC visit included CD4 count and viral suppression (defined as fewer than 50 copies of HIV RNA per milliliter of blood).

#### 2.2.2 Communication characteristics

As part of the customized netCanvas tool, participants were asked about their communication behaviors, including whether they had access to a smartphone and to describe how much time they spent on the phone (in hours) on a typical day.

#### 2.2.3 Ego-level network characteristics

Several ego-level network characteristics were included for each ego. *Network size* of each ego's network was defined as the number of alters identified by each ego. *Network density* of each ego was defined as the ratio of the number of edges an ego has over the number of possible edges the ego could have. Edges were defined if there were social connections (very close or spent time together in the past six months) between two alters. Network density ranges from 0 to 1, with lower values suggesting ego's alter network was more sparsely connected (i.e., fewer alters spend time with one another). *Emotional support size* was defined as the number of alters who were identified as providing emotional support to the ego.

#### 2.2.4 Alter-level characteristics

Participants described the relationship type with each alter (family/relative, friend, romantic partner, acquaintance, other). They also described how frequently they had seen each alter (rarely, weekly, daily), how close they felt to each alter (not very close, somewhat close, very close), how much they trusted each alter (not at all, not very much, somewhat, a lot), and how frequently they communicated with each alter by phone by talking or writing (never, rarely, weekly, daily). Participants also indicated whether each alter provided two types of instrumental support (i.e., they were prompted to “tap on all the people who have given money/given you a place to stay when you needed it”), one type of emotional support (i.e., tap on all the people with whom you have talked to about personal or private matters), and three types of medical support to the ego (i.e., “tap on all the people whom have provided you with information on medical services, helped you get to a medical appointment, or helped you take your medications”). Each type of social support was dichotomized for analysis. HIV disclosure to each alter was assessed with a prompt encouraging the participant to “tap on all the people to whom you have disclosed your HIV status.”

#### 2.2.5 Alter-level network characteristics

The following alter-level network measures were computed for all ego-alter ties. *Alter degree* was defined as the number of other alters an alter is connected to. *Alter harmonic centrality* was defined as the mean inverse distance from an alter to other alters. This measure ranges from 0 to 1, with a lower value indicating the alter is more isolated from the other alters.

### 2.3 Analytic approach

Descriptive statistics were utilized to describe both the characteristics of the egos and the ego-alter pairs. The unit of analysis was an ego-alter pair.

To evaluate the factors associated with disclosure, we formed a base model by considering the following variables: ego viral suppression (yes/no), ego gender, ego's knowledge of whether alter has HIV (yes/no), how much ego trusts alter (not at all, not very much, somewhat, a lot), age difference (alter age minus ego age), and main relationship type between ego and alter (family, friend, other). We first fit the base model with standard logistic regression to check for complete separation and collinearity among variables using the variance inflation factor. Based on the regression diagnostic, we combined the “not at all” and “not very much” response options for trust into one category.

We then considered adding network measures, including alter degree, alter harmonic centrality, emotional support size, medical support size, network density, and network size. This was done by fitting univariable logistic models and by adding each network measure to the base model. Generalized estimating equations (GEE) with robust standard errors and an exchangeable correlation structure for the ego-alter pairs accounting for clustering in egos were adopted in these exploratory models. The GEE framework was chosen over the generalized linear mixed model framework for interpretation reasons since interest was in population estimates of disclosure. For each of the network measures, the odds ratio (OR) from the univariable model and the adjusted odds ratio (AOR) from the multivariable model were estimated with 95% confidence intervals (CIs). The exploratory logistic GEE for emotional support size yielded counterintuitive results for emotional support size, which indicated a negative association of increasing emotional support size with disclosure. This result was counterintuitive because the size of the ego's emotional support network typically has a positive association with disclosure (30). Therefore, potential interactions were explored and an interaction effect of relationship type with emotional support size was identified. Based on the exploratory models, the final logistic GEE model was fit by adding alter harmonic centrality, network size, emotional support size, and interaction between emotional support size and relationship type to the base model.

All statistical analyses were performed in R 4.2.2 (R Core Team, Vienna, Austria). The *igraph* package was used to calculate the network measures, and the *geepack* package was used to fit GEE models (31, 32). All hypotheses were evaluated at the 0.05 Type-I level of significance. There was no adjustment for multiple testing as the analysis was exploratory.

## 3 Results

After excluding egos with missing relationship types, demographic information, or viral suppression status, the study included 58 egos with a total of 349 alters (network members) identified. The characteristics of the egos are presented in Table 1. Out of 58 egos, 52% (30/58) were female. The average age at the time of network measures assessment was 14.3 years [standard deviation (SD) 0.98], and approximately 66% (*n* = 38) were virally suppressed, with a median viral load of 39 copies/ml (SD 17,658.9 copies/ml). The mean CD4 count was 695.9 cells/μl (SD 244.5 cells/μl). The median network size of each ego was 5.5 individuals [first quartile (Q1) 4 and third quartile (Q3) 7], and network density was relatively low (mean 0.24, SD 0.28). Instrumental, emotional, and medical support network sizes were relatively small, with medians of 3, 2 and 2, respectively. Smartphone ownership or access was reported by 71% (*n* = 41) of participants; 24% (*n* = 14) reported no mobile access with the remaining either only having mobile access or missing values. Average cellphone usage time was 2.39 h per day (SD 2.21).

**Table 1:** Ego characteristics at the time of network measure assessment by gender.

Ego characteristics	Male (*N* = 28)	Female (*N* = 30)	Overall (*N* = 58)
Age			
Mean (SD)	14.4 (1.0)	14.2 (0.9)	14.3 (1.0)
Median (Q1, Q3)	14.5 (13.7, 15.1)	14.3 (13.6, 15.0)	14.4 (13.7, 15.1)
[Min, Max]	[12.5, 16.3]	[12.1, 15.5]	[12.1, 16.3]
Viral load (copies/ml)			
Mean (SD)	3,793.3 (17,658.9)	1,198.7 (2,924.7)	2,451.2 (12,400.6)
Median (Q1, Q3)	39 (39, 100)	39 (39, 100)	39 (39, 100)
[Min, Max]	[34, 93,217]	[39, 12,541]	[34, 93,217]
Virally suppressed			
Not virally suppressed	9 (32%)	11 (37%)	20 (34%)
Virally suppressed	19 (68%)	19 (63%)	38 (66%)
CD4 count (cells/μl)			
Mean (SD)	626 (206.8)	761.1 (261.9)	695.9 (244.5)
Median (Q1, Q3)	663.5 (490.5, 752)	760.5 (608, 928)	703 (513, 812.3)
[Min, Max]	[159, 1,049]	[139, 1,300]	[139, 1,300]
Network size			
Mean (SD)	5.4 (2.8)	6.6 (4.5)	6.0 (3.8)
Median (Q1, Q3)	5 (3.8, 6.3)	6 (4, 7.8)	5.5 (4, 7)
[Min, Max]	[2, 13]	[2, 22]	[2, 22]
Network density			
Mean (SD)	0.27 (0.32)	0.21 (0.24)	0.24 (0.28)
Median (Q1, Q3)	0.15 (0.07, 0.33)	0.14 (0.10, 0.20)	0.14 (0.07, 0.33)
[Min, Max]	[0, 1]	[0, 1]	[0, 1]
Instrumental support network size			
Mean (SD)	3.6 (2.1)	4.7 (3.9)	4.2 (3.2)
Median (Q1, Q3)	3 (2.8, 4)	3.5 (2.3, 5)	3 (2.3, 4.8)
[Min, Max]	[1, 10]	[0, 17]	[0, 17]
Emotional support network size			
Mean (SD)	2.6 (1.8)	2.2 (2.1)	2.4 (1.9)
Median (Q1, Q3)	2 (1, 3)	2 (1, 3)	2 (1, 3)
[Min, Max]	[1, 8]	[0, 11]	[0, 11]
Medical support network size			
Mean (SD)	2.5 (1.4)	3.3 (2.9)	3.0 (2.3)
Median (Q1, Q3)	2 (1, 4)	2.50 (2, 4)	2 (2, 4)
[Min, Max]	[1, 6]	[0, 15]	[0, 15]
Smartphone access			
Owns smartphone	18 (64%)	17 (57%)	35 (60%)
Does not own smartphone but has smartphone access	3 (11%)	3 (10%)	6 (10%)
Has mobile access but not smartphone access	1 (4%)	0 (0%)	1 (2%)
No mobile access	5 (18%)	9 (30%)	14 (24%)
Missing	1 (4%)	1 (3%)	2 (3%)
Cellphone time (hours)			
Mean (SD)	2.8 (2.4)	2 (2)	2.4 (2.2)
Median (Q1, Q3)	2 (1.5, 4)	2 (0, 3)	2 (0.75, 3)
[Min, Max]	[0, 10]	[0, 8]	[0, 10]
Missing	1 (3.6%)	1 (3.3%)	2 (3.4%)

Of 58 egos, 86% (*n* = 50) had disclosed their HIV status to at least one individual. However, the percentage of alters each ego disclosed to within their network was a median of 33.3%, (Q1 20%, Q3 63%). Table 2 displays alter characteristics, stratified by disclosure. Out of 349 alters, 36.4% (127/349) were disclosed to by an ego. Overall, the majority of alters were family or relatives (66.8%), with friends comprising 23.5%. A higher proportion of disclosed alters were family members (90.6%) compared to not disclosed alters (53.2%), while a lower proportion of disclosed alters were friends (6.3%) compared to not disclosed alters (33.3%). Alters who were disclosed to were also older on average (mean age 37.4, SD 15.7 years) than those who were not disclosed to (mean age 23.4, SD 14.1 years). Disclosed alters were more frequently described as “very close” (76.4%) and trusted “a lot” (75.6%) compared to alters who were not disclosed to (50.9% and 44.1%, respectively). Additionally, disclosed alters were much more likely to provide instrumental (89% vs. 57.7%), emotional (52.8% vs. 32%), and medical support (75.6% vs. 33.8%) than alters who were not disclosed to.

**Table 2:** Alter characteristics by disclosure.

Alter characteristics	Not disclosed to	Disclosed to	Overall
	(*N* = 222)	(*N* = 127)	(*N* = 349)
Alter Age			
Mean (SD)	23.4 (14.1)	37.4 (15.7)	28.5 (16.1)
Median (Q1, Q3)	16 (14, 30)	40 (25, 46)	24 (14, 41)
[Min, Max]	[6, 78]	[7, 90]	[6, 90]
Alter age minus ego age			
Mean (SD)	9.1 (14.1)	23.2 (15.7)	14.3 (16.2)
Median (Q1, Q3)	1.7 (−0.6, 15.5)	25.0 (10.6, 31.8)	10.1 (0.1, 26.1)
[Min, Max]	[−8.7, 64.4]	[−6.8, 76.5]	[−8.7, 76.5]
Relationship type			
Family/relative	118 (53.2%)	115 (90.6%)	233 (66.8%)
Friend	74 (33.3%)	8 (6.3%)	82 (23.5%)
Romantic partner	9 (4.1%)	1 (0.8%)	10 (2.9%)
Acquaintance	6 (2.7%)	0 (0%)	6 (1.7%)
Other support	15 (6.8%)	3 (2.4%)	18 (5.2%)
How frequently have you seen?			
Rarely	45 (20.3%)	12 (9.4%)	57 (16.3%)
Weekly	66 (29.7%)	31 (24.4%)	97 (27.8%)
Daily	111 (50.0%)	84 (66.1%)	195 (55.9%)
How close to you feel?			
Not very close	36 (16.2%)	4 (3.1%)	40 (11.5%)
Somewhat close	73 (32.9%)	26 (20.5%)	99 (28.4%)
Very close	113 (50.9%)	97 (76.4%)	210 (60.2%)
Trust			
Not at all	9 (4.1%)	1 (0.8%)	10 (2.9%)
Not very much	41 (18.5%)	8 (6.3%)	49 (14.0%)
Somewhat	74 (33.3%)	22 (17.3%)	96 (27.5%)
A lot	98 (44.1%)	96 (75.6%)	194 (55.6%)
Phone talk frequency			
Never	95 (42.8%)	58 (45.7%)	153 (43.8%)
Rarely	36 (16.2%)	23 (18.1%)	59 (16.9%)
Weekly	56 (25.2%)	15 (11.8%)	71 (20.3%)
Daily	35 (15.8%)	31 (24.4%)	66 (18.9%)
Phone written frequency			
Never	97 (43.7%)	60 (47.2%)	157 (45.0%)
Rarely	30 (13.5%)	23 (18.1%)	53 (15.2%)
Weekly	43 (19.4%)	18 (14.2%)	61 (17.5%)
Daily	52 (23.4%)	26 (20.5%)	78 (22.3%)
Provided instrumental support			
No	94 (42.3%)	14 (11.0%)	108 (30.9%)
Yes	128 (57.7%)	113 (89.0%)	241 (69.1%)
Provided emotional support			
No	151 (68.0%)	60 (47.2%)	211 (60.5%)
Yes	71 (32.0%)	67 (52.8%)	138 (39.5%)
Provided medical support			
No	147 (66.2%)	31 (24.4%)	178 (51.0%)
Yes	75 (33.8%)	96 (75.6%)	171 (49.0%)
Alter degree			
0	116 (52.3%)	38 (29.9%)	154 (44.1%)
1	88 (39.6%)	78 (61.4%)	166 (47.6%)
2	11 (5.0%)	10 (7.9%)	21 (6.0%)
3	7 (3.2%)	1 (0.8%)	8 (2.3%)
Alter betweenness			
Mean (SD)	0.15 (0.67)	0.15 (0.64)	0.15 (0.65)
Median (Q1, Q3)	0 (0, 0)	0 (0, 0)	0 (0, 0)
[Min, Max]	[0, 6]	[0, 5.33]	[0, 6]
Alter harmonic centrality			
Mean (SD)	0.12 (0.17)	0.25 (0.28)	0.17 (0.23)
Median (Q1, Q3)	0 (0, 0.2)	0.2 (0, 0.33)	0.11 (0, 0.25)
[Min, Max]	[0, 1]	[0, 1]	[0, 1]

The results from the univariable and multivariable GEE logistic models examining the relationships between network measures and disclosure are summarized in Table 3. Univariable analyses suggested significant positive associations of alter harmonic centrality (OR 1.03, 95% CI: 1.01–1.04, *p* < 0.001) and network density (OR 1.03, 95% CI: 1.01–1.05, *p* < 0.001) with disclosure. These associations remained significant in the multivariable models, though the effects were slightly attenuated. After controlling for base variables, a 0.01 increase in alter harmonic centrality was associated with a 2% increase in the odds of disclosure (AOR 1.02, 95% CI: 1.002–1.03, *p* = 0.02), while a 0.01 increase in network density is associated with a 2% increase in the odds of disclosure (AOR 1.02, 95% CI: 1–1.039, *p* = 0.045). Emotional support network size was not associated with disclosure in the univariable model (OR 0.89, 95% CI: 0.76–1.04, *p* = 0.13), but it was significantly associated in the multivariable model; in the multivariable model, each additional alter who provided emotional support was associated with a 26% decrease in the odds of disclosure (AOR 0.74, 95% CI: 0.57–0.96, *p* = 0.02). This means that on average for two groups who are alike for all variables except for emotional support size, the group with a smaller emotional support size has a higher odds of being disclosed to than a group with a larger emotional support size. Other network measures, including alter degree, medical support size, and network size, did not show significant associations in either univariable or multivariable models.

**Table 3:** Univariable and multivariable GEE logistic regression results for each network measure of interest.

Network measures	Unadjusted odds ratio (95% CI)	Adjusted odds ratio^a^ (95% CI)
Alter degree (1-degree increase)	1.62 (0.94, 2.78, *p* = 0.08)	1.37 (0.80, 2.34, *p* = 0.26)
Alter harmonic centrality (0.01 increase)	1.03 (1.01, 1.04, *p* < 0.001)	1.02 (1.002, 1.03, *p* = 0.02)
Emotional support size (1-alter increase)	0.89 (0.76, 1.04, *p* = 0.13)	0.74 (0.57, 0.96, *p* = 0.02)
Medical support size (1-alter increase)	0.91 (0.8, 1.03, *p* = 0.12)	1.03 (0.82, 1.28, *p* = 0.82)
Network density (0.01 increase)	1.03 (1.01, 1.05, *p* < 0.001)	1.02 (1, 1.04, *p* = 0.045)
Network size (1-alter increase)	0.91 (0.80, 1.02, *p* = 0.11)	1.02 (0.88, 1.18, *p* = 0.78)

^a^ Each multivariable GEE logistic regression model included the following covariates plus the network measure of interest: ego viral suppression (Yes/No), ego gender, ego's knowledge of whether alter has HIV (Yes/No), how much ego trusts alter (not at all or not very much, somewhat, a lot), age difference (alter age minus ego age), and main relationship type between ego and alter (family, friend, other).

**Figure 2:**
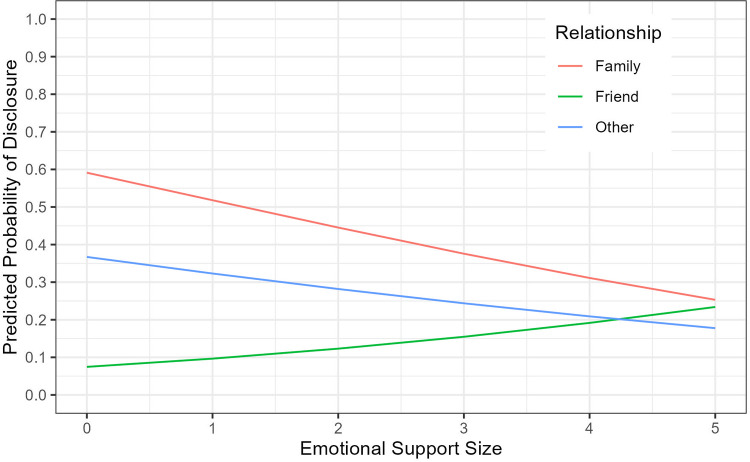
Estimated probabilities of disclosure by emotional support size and relationship from the final GEE logistic regression model.

After observing a negative association of emotional support size with disclosure, an interaction of emotional support size with relationship type was included. The results of the final GEE logistic model are summarized in Table 4, which suggest that the odds of disclosure did not differ based on the ego's gender or viral suppression. The odds of disclosure was significantly greater when alters were presumed by the ego to have HIV (AOR 6.26, 95% CI: 2.36–16.58, *p* < 0.001), trusted a lot by the ego (AOR 2.84, 95% CI: 1.14–7.06 *p* = 0.03), or older than the ego (1-year difference AOR 1.04, 95% CI: 1.02–1.07, *p* < 0.001). Increasing alter's centrality by 0.01 was associated with an increased odds of disclosure (AOR 1.02; 95% CI: 1.001–1.03, *p* = 0.03), though overall network size was not associated with disclosure. The effect of the ego's emotional support network size on disclosure, however, depended on the type of ego-alter relationship. If the alters were family members, the odds of disclosure decreased by 30% as the emotional support size increased by 1 (AOR 0.70, 95% CI: 0.54–0.9, *p* = 0.006). If the alters were friends, the odds of disclosure increased by 32% as the emotional support size increased by 1 (AOR 1.32, 95% CI: 1.05–1.65, *p* = 0.016). The emotional support size was not associated with disclosure when the alters were other relationships. Estimated probabilities of disclosure by emotional support size and relationship from the final GEE logistic regression model are visualized in Figure 2.

**Table 4:** Adjusted odds ratios from the final GEE logistic regression model.

Variables	Adjusted odds ratio (95% CI)
Viral suppression: No	Reference
Viral suppression: Yes	0.84 (0.37, 1.93, *p* = 0.68)
Ego gender: Male	Reference
Ego gender: Female	0.64 (0.27, 1.55, *p* = 0.32)
Knowledge that Alter is HIV+: No	Reference
Knowledge that Alter is HIV+: Yes	6.26 (2.36, 16.58, *p* < 0.001)
Trust (Not at all or Not very much)	Reference
Trust (Somewhat)	2.12 (0.85, 5.27, *p* = 0.11)
Trust (A lot)	2.84 (1.14, 7.06, *p* = 0.03)
Alter-ego age difference (1 year difference)	1.04 (1.02, 1.07, *p* < 0.001)
Alter harmonic centrality (0.01 increase)	1.02 (1.002, 1.033, *p* = 0.03)
Network size (1-alter increase)	1.08 (0.97, 1.19, *p* = 0.154)
Relationship type is Friend vs. Family (reference) when emotional support size = 1	0.06 (0.01, 0.26, *p* < 0.001)
Relationship type is Other vs. Family (reference) when emotional support size = 1	0.25 (0.04, 1.50, *p* = 0.13)
Relationship type is Friend vs. Family (reference) when emotional support size = 2	0.11 (0.03. 0.40, *p* = 0.001)
Relationship type is Other vs. Family (reference) when emotional support size = 2	0.30 (0.06, 1.42, *p* = 0.13)
Relationship type is Friend vs. Family (reference) when emotional support size = 3	0.20 (0.06, 0.65, *p* = 0.007)
Relationship type is Other vs. Family (reference) when emotional support size = 3	0.36 (0.09, 1.42, *p* = 0.14)
Emotional support size increases by 1 when relationship type is Family	0.70 (0.54, 0.9, *p* = 0.006)
Emotional support size increases by 1 when relationship type is Friend	1.32 (1.05, 1.65, *p* = 0.02)
Emotional support size increases by 1 when relationship type is Other	0.84 (0.62, 1.13, *p* = 0.24)

## 4 Discussion

The majority (86%) of the adolescents in our study had disclosed their HIV status to at least one person, which is higher than prior studies, which have reported rates of disclosure among adolescents to at least one person from 33% to 66% (11, 15, 33, 34). One study (11) noted that their participants may have interpreted questions on family disclosure to refer to extended family members and not immediate family members. Our approach to first enumerating all current network members, including immediate family members such as biological parents and caregivers, and then asking about disclosure to each individual named was unlikely to have had this issue, potentially explaining the higher levels of disclosure to at least one network member in our study.

HIV disclosure across all social ties within APHIV's social networks was only 36.4%. This relatively low level of disclosure is consistent with previous research that has highlighted the challenges young people face with disclosure (12, 35). For example, a recent systematic review grounded in the Disclosure Process Model (36), which aims to understand why and when people disclose their status, found that anticipated fear and stigma were the most common reason reported for failure to disclose (37). Given the limited levels of disclosure within networks, interventions promoting safe and effective disclosure in this setting may benefit from interventions that support internalized HIV stigma reduction and stigma resistance as well as broader community-level stigma reduction, both of which have been recommended by other disclosure researchers.

Adolescents were more likely to disclose to individuals they trust, perceive as having HIV, or who are older and more central in their social networks. These findings are consistent with previous egocentric network analysis with African American people living with HIV, who were more likely to have disclosed to others who were highly trusted, had a higher level of centrality, and who were also living with HIV (38). Given the sensitive nature of HIV disclosure, it is reasonable that adolescents may feel more comfortable disclosing to individuals they trust. However, due to the cross-sectional design of this study, it is also possible that trust was strengthened following disclosure or that disclosure influenced subsequent perceptions of a network member's trustworthiness. Additionally, network members who are also living with HIV may be better-able to relate and to provide support and guidance than others in the network. Additionally, those who are centrally located within the network may be better positioned to connect the adolescent to others to provide support (38). As such, these findings may reflect adolescents’ efforts to navigate stigma and power within their networks. Disclosing to older and/or more central individuals could connect the adolescents with gatekeepers or advocates who are well-positioned to influence attitudes and norms within their social network.

We also found that disclosed network members were much more likely to provide various types of social support than those who were not disclosed to. These findings are consistent with other studies that have found that individuals are most likely to disclose to family members or close friends (39) and that people with HIV may be often motivated to disclose in order to obtain tangible social support, including transportation to medical appointments or and reminders for treatment adherence (39). These findings highlight the multifaceted nature of disclosure, where the act of disclosing can be both a means to access support as well as an opportunity to deepen social relationships.

While overall network size was not associated with disclosure, the type of relationship between the adolescent and the network member moderated the effect of emotional support network size. Larger emotional support networks were associated with a decreased odds of disclosure to family members but an increased odds of disclosure to friends. These results underscore the potential importance of considering both individual and relational factors when designing interventions to support safe and effective disclosure strategies among APHIV, tailored to the unique social contexts of family and peer relationships. The results also suggest that empowering peers with skills to effectively provide emotional support to each other may serve to increase disclosure in this population.

Our study highlights the benefits and future potential of using social network data collection and analysis methods to better understand disclosure. By using a tablet-based data collection method, we were able to efficiently assess key factors of the adolescent's relationship to multiple individuals. This approach would be well-suited to exploring additional ego-alter characteristics, such as disclosure concerns, characteristics of the disclosure process, and outcomes of disclosure with each alter. Recent research noting the importance of disclosure concerns, for example, highlighted that the effect of disclosure concerns on ART adherence and viral suppression likely depends on which individual(s) are a source of disclosure concerns (40).

Additionally, social network data collection and analysis methods may also be particularly relevant as APHIV continue to grow into young adulthood and engage in sexual relationships. HIV status disclosure has historically been a key component of efforts to prevent the onward transmission of HIV (41). Disclosure has been seen as key to the negotiation of safer sexual behavior, including condom use, and a gateway to engaging partners in HIV testing and care, including pre-exposure prophylaxis (PrEP) for partners without HIV and ART for partners with HIV (42). However, research with adolescents has not found a relationship between disclosing one's HIV status to a partner with safer sexual behavior (11, 33, 43). While HIV disclosure has the potential to lead to the negotiation of safe sexual practices or open pathways to care, it simultaneously requires individuals to confront stigma, navigate complex power dynamics, and be equipped to negotiate safer sex. Using social network analysis methods to explore these dynamics within relationships may be particularly useful, especially in the context of U = U (Undetectable = Untransmittable), which demonstrates that individuals with undetectable viral loads cannot sexually transmit HIV. Recent qualitative research has found that some people with suppressed viral loads feel as though disclosing their HIV status, particularly to sexual partners, is a shared responsibility and that partners should inquire about their HIV status (44). Collecting data on this social context of disclosure (i.e., assessing whether each network member initiated the HIV disclosure process with the adolescent) using tablet-based approaches to network data collection can facilitate our understanding of this topic in the South African context, where awareness of U = U among adolescents and adults with HIV remains low (45, 46).

The findings of our study should be understood within the context of study limitations. The egocentric network data collected in this study were based on the APHIV's self-report and as such, could be biased by the adolescent's perception of social ties between network members. Also, while the use of the tablet-based data collection tool reduced participant burden, there are limitations specific to the way this tool measured binary ego-alter characteristics, like disclosure (and alter's HIV status). This was done by prompting the participant to tap on all the individuals in the network to whom they disclosed (or whom they perceived to be living with HIV). This means that any alters that were not selected by the participant were presumed to be intentionally omitted rather that overlooked unintentionally by the ego. Additionally, the study sample was limited to 58 egos and the sample was recruited from HIV clinics, so the results presented may not generalize to other APHIV, particularly those that are not engaged in HIV care. Also, the findings apply to adolescents who are well established on ART and who's HIV has been well controlled; they may not be applicable to those not on ART or with less well controlled HIV. Finally, the study is hypothesis-generating and exploratory in its nature and therefore did not account for multiple testing.

Findings from this research could have implications for designing interventions that support safe and effective disclosure practices, ultimately enhancing the health and well-being of this population. Network members to whom adolescents have disclosed can potentially impact the adolescents’ treatment adherence and HIV care engagement, and these individuals could be engaged to provide critical social support to the adolescents. These individuals could be explicitly engaged in interventions to promote disclosure as well. For example, a recent feasibility trial testing a 4-session group intervention to promote disclosure among young adults with perinatally-acquired HIV found that outlining external support options would be beneficial (47). Network members to whom adolescents have already disclosed could be leveraged for this role. Our findings also speak to the need for tailored interventions that acknowledge the complexities of disclosure decisions to help APHIV navigate these challenges while promoting autonomy in their disclosure journeys. Given that APHIV are more likely to disclose to trusted individuals and those perceived to be living with HIV, interventions could focus on strengthening social support networks and empowering adolescents with strategies to safely disclose to key support figures. Additionally, our findings emphasize the importance of equipping healthcare providers with the necessary training to address disclosure concerns sensitively. Healthcare workers can play a crucial role in supporting adolescents through the disclosure process by providing guidance, addressing stigma-related fears, and facilitating discussions around disclosure within families and peer networks. Healthcare workers may be particularly well-positioned to provide this support, as they often play a role in informing APHIV of their HIV status (48). Other studies have identified the need for better support at schools, including policies to facilitate disclosure and offer post-disclosure support (49). Finally, community-based programs may offer an important avenue for fostering supportive social environments. Programs that work to reduce HIV stigma at the community level and enhance peer support networks could help create safer spaces for disclosure and, in turn, improve adolescents’ engagement in HIV care. Future research should explore how integrating social network-based approaches into community and clinical settings may further facilitate safe and empowering disclosure practices among adolescents with HIV.

In conclusion, this study highlights the potential role of social networks in shaping HIV disclosure among APHIV. While most participants disclosed their status to at least one person, overall disclosure within social networks remained low, highlighting the need for interventions that address both individual and structural barriers to disclosure. Strengthening trusted relationships and addressing community stigma are essential components of disclosure support strategies. To build on these findings, researchers should employ longitudinal network analysis to examine disclosure within the social context of dynamic relationships. Studies assessing the effectiveness of peer-led disclosure interventions and community stigma-reduction efforts would also provide valuable insights. Policymakers should prioritize initiatives that ensure APHIV receive tailored disclosure support through clinic and community settings. Finally, healthcare providers should receive training in disclosure counseling, stigma reduction strategies, and family engagement approaches to better support APHIV in their disclosure journeys.

## Data Availability

The datasets presented in this article are not readily available because the potential for heightened risk of reidentification of certain data and the sensitive topic of the research (HIV disclosure among adolescents living with HIV). Data may be made available through a data use agreement. Requests to access the datasets should be directed to Marta Mulawa, marta.mulawa@duke.edu.
